# CADENCE: Open-source software for signal conditioning, feature extraction, visualization and analysis of cardiac optical mapping data

**DOI:** 10.64898/2026.09.30.755844

**Published:** 2026-10-07

**Authors:** Kedar Aras, Shaivi Pandey, Adedayo Adelakun, Ethan Minnella, Alexis Lichtenberger, Neelam Qadus, Luke Barnhart

## Abstract

Optical mapping is a fundamental tool for cardiac electrophysiology research. Processing of these signals can be non-trivial, requiring multiple pre- and post-processing steps to limit interpretation errors. Existing software packages have features siloed across platforms and require users to combine several packages for in-depth analysis. To address this, we developed CADENCE, an open-source MATLAB-based application combining feature breadth (e.g., SVD denoising, conduction velocity, alternans, annotated rotors) and depth (e.g., 15 independent alternans features across both voltage and calcium modalities) in an end-to-end pipeline including data conversion, signal conditioning, feature extraction, and signal analysis. We demonstrate CADENCE’s capabilities across datasets spanning four species (rat, rabbit, pig, and human), three tissue preparations (slices, wedge, and whole heart), and three camera configurations (single, dual, and panoramic). CADENCE was validated against three blinded expert observers (15 hearts) and demonstrated agreement comparable to inter-observer variability (SD ratios 1.07/0.95/0.89 for APD80, activation, CaTD80). Dominant frequency was recovered with a median error of 0.03% across 84 of 88 paced recordings with 1:1 capture. CADENCE’s code is publicly available and distributed as a compiled standalone application that the end-user can install and run without a MATLAB license to encourage adoption and standardization across laboratories.

## Full Text Availability

The license terms selected by the author(s) for this preprint version do not permit archiving in PMC. The full text is available from the preprint server.

